# Quality Improvement Initiatives to Assess and Improve PET/CT Injection Infiltration Rates at Multiple Centers

**DOI:** 10.2967/jnmt.119.228098

**Published:** 2019-12

**Authors:** Terence Z. Wong, Thad Benefield, Shane Masters, Jackson W. Kiser, James Crowley, Dustin Osborne, Osama Mawlawi, James Barnwell, Pawan Gupta, Akiva Mintz, Kelley A. Ryan, Steven R. Perrin, Ronald K. Lattanze, David W. Townsend

**Affiliations:** 1 Duke University, Durham, North Carolina; 2 University of North Carolina, Chapel Hill, North Carolina; 3 Wake Forest Baptist Medical Center, Winston Salem, North Carolina; 4 Carilion Clinic, Roanoke, Virginia; 5 Radiology/Molecular Imaging and Translational Research, University of Tennessee Graduate School of Medicine, Knoxville, Tennessee; 6 Department of Imaging Physics, M.D. Anderson Cancer Center, University of Texas, Houston, Texas; 7 Wake Radiology, Raleigh, North Carolina; 8 Division of Nuclear Medicine, Department of Molecular and Medical Pharmacology, David Geffen School of Medicine at UCLA Health, Los Angeles, California; 9 Columbia University Medical Center, New York, New York; 10 Lucerno Dynamics, LLC, Cary, North Carolina; and; 11 A*STAR-NUS Clinical Imaging Research Centre, Singapore

**Keywords:** quality improvement, PET/CT, infiltration, extravasation, FDG

## Abstract

PET/CT radiotracer infiltration is not uncommon and is often outside the imaging field of view. Infiltration can negatively affect image quality, image quantification, and patient management. Until recently, there has not been a simple way to routinely practice PET radiopharmaceutical administration quality control and quality assurance. Our objectives were to quantify infiltration rates, determine associative factors for infiltration, and assess whether rates could be reduced at multiple centers and then sustained. **Methods:** A “design, measure, analyze, improve, and control” quality improvement methodology requiring novel technology was used to try to improve PET/CT injection quality. Teams were educated on the importance of quality injections. Baseline infiltration rates were measured, center-specific associative factors were analyzed, team meetings were held, improvement plans were established and executed, and rates remeasured. To ensure that injection-quality gains were retained, real-time feedback and ongoing monitoring were used. Sustainability was assessed. **Results:** Seven centers and 56 technologists provided data on 5,541 injections. The centers’ aggregated baseline infiltration rate was 6.2% (range, 2%–16%). On the basis of their specific associative factors, 4 centers developed improvement plans and reduced their aggregated infiltration rate from 8.9% to 4.6% (*P* < 0.0001). Ongoing injection monitoring showed sustainability. Significant variation was found in center- and technologist-level infiltration rates (*P* < 0.0001 and *P* = 0.0020, respectively). **Conclusion:** A quality improvement approach with new technology can help centers measure infiltration rates, determine associative factors, implement interventions, and improve and sustain injection quality. Because PET/CT images help guide patient management, the monitoring and improvement of radiotracer injection quality are important.

An estimated 3 million PET/CT procedures were performed in the United States in 2017, with over 90% being for oncology care and approximately 10% for assessing myocardial perfusion, neurologic function, and other physiologic processes (1,2). Complete delivery of an intravenous bolus of radiotracer is important to the accuracy and reproducibility of imaging (3) and thus to patient treatment (4). A radiotracer infiltration prevents a bolus delivery of the entire dose. Infiltration happens when a catheter punctures or erodes the venous wall or when injection pressure damages the wall, leading to infusion of fluid into the soft tissue surrounding the vein. The severity of the effect on image quality and quantification cannot be determined precisely (4) but depends on the initial infiltrate amount, the rate at which infiltrate reenters circulation, and the residual infiltrate amount that never enters circulation.

Unlike other health-care injection processes that monitor injection quality (e.g., contrast-enhanced CT and chemotherapy) (5–7), there is no evidence that PET/CT injections are routinely monitored. Difficulty in detection may be a factor. PET/CT technologists usually inject small radiotracer volumes that do not cause immediate patient pain and rarely cause visible changes to the skin near the injection site. Furthermore, during PET/CT image interpretation, the injection site is often outside the imaging field of view (8). Detection is further hindered when the injection site is within the imaging field of view but the infiltration has resolved completely, leaving no visible evidence (9). There are also few published data on PET/CT radiotracer injection infiltration rates. A literature review identified 6 studies (2006–2017) from 3 centers that used routine static images as their method to identify infiltration. These studies involved 2,804 patients and 425 infiltrations (15.2%). Rates ranged from 3% to 23% (8,10–14) and, on the basis of detection difficulties, may have underestimated true infiltration rates (9).

Our hypotheses were that a quality improvement (QI) approach could measure infiltration rates for patients undergoing PET/CT examinations across multiple centers, determine associative factors that may contribute to infiltration, and measure the reduction in rates of infiltration.

## MATERIALS AND METHODS

An Institutional Review Board for each center determined that the project did not meet the definition of research as defined by the federal government in title 45 of *Code of Federal Regulations,* part 46.102(d); therefore, no patient consent was required. No protected health information was collected.

Because QI approaches have led to high-quality results for chemotherapy and contrast-CT injections (6,7) in patient populations such as those undergoing PET/CT radiotracer injections, we believed that following a QI process for PET/CT could lead to similar results. The “define, measure, analyze, improve, control” QI methodology was used.

In the define phase, the infiltration problem, injection process, clinician/center needs, and potential factors associated with infiltration were defined in a protocol approved by each center. Seven centers participated on the condition of anonymity and aggregation of data. Centers were sequentially initiated from December 2016 to July 2017 (approximately 1 center/mo). Centers included 2 low-volume (<2 patients/d) outpatient or mobile units, a medium-volume (∼5 patients/d) community care hospital, 3 high-volume (∼18 patients/d) academic centers, and a very high-volume (>30 patients/d) cancer care center. Before center initiation, 56 certified nuclear medicine technologists (experience ranging from 1–41 y; mean, 13.8 y; median, 12.5 y), 5 nuclear medicine physicians, and 2 physicists participated and were educated on the project and on the importance of the injection process.

**FIGURE 1.**
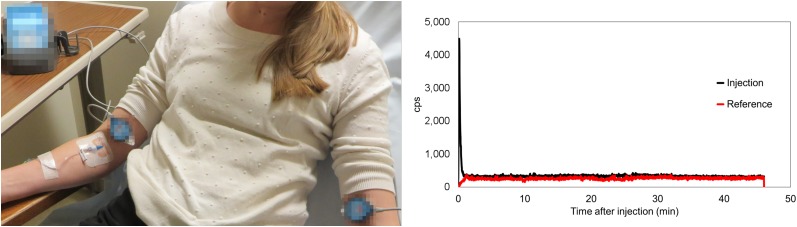
Lara system consists of 2 scintillation sensors, 2 pads, reader, and docking station. Sensors are placed on injection arm and contralateral arm. Time–activity curve is provided after data upload.

Because nuclear medicine injection quality is not routinely measured, an infiltration detection method was needed to consistently determine baseline performance across centers. Therefore, novel technology was required in the measure phase. A commercially available system, Lara (Lucerno Dynamics), was selected on the basis of clinical studies demonstrating its ability to identify the presence of radiotracer near the injection site and to help reduce infiltration rates (9,13,15). The Lara system includes topical sensors and a reader to collect and store data, software to transfer data, and a web application to display and analyze data. Use of the system adds about 30 s to the patient experience and 90 s to the technologist experience. The system assists clinicians in assessing injection quality by providing injection-arm and reference-arm time–activity curves during the uptake period (Fig. 1). Time–activity curves are scored by an automated classifier, developed from injections that had been qualitatively evaluated by nuclear medicine physicians.

In the measure phase, technologists used the system to monitor radiotracer injections for adult and pediatric patients for 2–4 mo, based on center volume. After gaining venous access, and before injecting patients with a radiotracer, technologists applied atraumatic adhesive pads and then sensors to the patient. One pad/sensor was applied approximately 7 cm proximal to the injection site; another was applied in the mirrored location on the contralateral arm. Data were recorded by the system during the tracer uptake period (typically 45–60 min). After removing the pads/sensors from the patient, technologists uploaded patient-specific (height, weight, body mass index, glucose level, age group [<16, 16–49, 50–69, >70 y]) and procedure-specific (injecting technologist, venous access method, radiotracer dose, flush volume, needle gauge, injection site location and orientation [right or left]) variables to the system’s web application. Time–activity curves were immediately generated. During this phase, the time–activity curves were not made available to technologists so that the review would not influence technologist technique. Time–activity curves were independently assessed by the system developer. Scores higher than 200 were considered to indicate the presence of radiotracer at the injection site. Scores higher than 1,000 were communicated to the principal investigators of the center to ensure that the interpreting physicians would be aware of potential patient care implications caused by the presence of radiotracer near the injection site. Reimaging and assessment of the potential clinical effect of radiotracer at the injection site were outside the scope of the project. Weekly use data (number of time–activity curves compared with number of PET/CT patients) were collected, analyzed, and reported to the centers to encourage them to use the Lara system. After this phase and throughout the remainder of the project, technologists received time–activity curve injection feedback immediately after uploading data.

The analyze phase began with group-level team meetings at each center. The team reviewed and discussed the use rates and time–activity curves. Principal investigators confirmed the measured infiltration rates. The Lara system provided center-specific insight into potential factors associated with poor-quality injections by analyzing patient- and procedure-specific variables collected from measure-phase injections.

Four centers proceeded to the improve phase; each held brainstorming sessions and created specific improvement plans based on associative factors and injection improvement interventions and ideas (Supplemental Table 1; supplemental materials are available at http://tech.snmjournals.org/). After the improvement plans were implemented and injection practices modified, the centers remeasured rates by monitoring a similar number of injections by the same measure-phase technologists. At the end of the improve phase, use rates, time–activity curves, infiltration rates, and adherence to improvement plans were evaluated.

After completing their improve phase, 3 centers monitored injections for an extended period to the assess the sustainability of injection QI in the control phase while the fourth center completed its improve phase. Ongoing group-level and individual-level feedback was provided in real time during this phase. Documentation of qualitative performance feedback for each technologist was outside the scope of this project. Overall project data collection ceased at all centers when the fourth center completed its improve phase. After completion of the project, all 4 centers continued to monitor injection quality to ensure routine quality control and quality assurance.

### Statistical Methods

#### Coprimary Endpoints

The first coprimary endpoint was the aggregated infiltration rate across measure-phase centers. Unadjusted rates were calculated by dividing the total number of infiltrations (for all centers) by the total number of injections. Adjusted rates were calculated using a multilevel generalized linear mixed model accounting for technologist-, center-, and patient-level correlations. The second coprimary endpoint was the aggregated adjusted rate of reduction in infiltration rates (aggregated measure-phase rate minus aggregated improve-phase rate) across the improve-phase centers. The *P* value for the test of H_0_ (no difference between the improve-phase and measure-phase adjusted infiltration rates) was reported.

#### Secondary Endpoints

There were 4 secondary endpoints: identify associative factors most likely to lead to infiltration, evaluate each improve-phase center’s infiltration rate reduction, assess each center’s adherence to the improvement plan, and evaluate variations in infiltration rate at the technologist or center level.

To identify associative factors most likely to lead to infiltration, aggregated data gathered during the measure, improve, and control phases were used to assess associations with injection quality. Main effects (patient- and procedure-specific variables), along with possible 2-way interactions, were evaluated (Supplemental Table 2).

To evaluate the rate of reduction of infiltration, centers needed to complete the analyze and improve phases. Binary decision trees and logistic regression were used to assess candidate covariate associations with injection quality during the analyze phase (Supplemental Table 3). The percentage infiltration rate reduction for improve-phase centers was defined as 100 × [(improve-phase rate − measure-phase rate)/measure-phase rate].

To estimate each center’s adherence to the improvement plan, interventions were categorized as a one-time or ongoing activity. On the basis of intervention adherence and its ability to affect injection quality, a center’s qualitative overall adherence to a proposed improvement plan was estimated (Supplemental Table 1).

To evaluate variations in infiltration rate at the center or technologist level, a likelihood ratio test using the pseudolikelihood was conducted on data from all phases and centers.

#### Exploratory Analysis

To assess improvement plan sustainability, differences were tested between the control- and measure-phase infiltration rates and between the control- and improve-phase rates. *P* values were adjusted using the Tukey method to control for type 1 error.

## RESULTS

Data were collected on 5,541 injections: 2,429 measure-phase injections, 1,349 improve-phase injections, and 1,763 control-phase injections. Measure-phase use of the Lara device ranged from 30% to 99% (mean and median use, 91% and 93%, respectively). Improve-phase use ranged from 85% to 93% (mean and median use, 90% and 91%, respectively). Technologist infiltration rates ranged from 0% to 24.4%.

### Coprimary Endpoints

The aggregated unadjusted infiltration rate for the 7 measure-phase centers was 6.2% (range, 1.9%–15.7%) (Table 1). The aggregated adjusted infiltration rate was 5.7% (SE, 1.8%; 95% confidence interval, 3.0%–10.6%)

**TABLE 1:** Unadjusted Measure-Phase Infiltration Rates

Center	Rate
A	13.3%
B	15.7%
C	12.8%
D	2.1%
E	3.2%
F	2.7%
G	1.9%

Centers’ volumes are not included to ensure center anonymity.

Measure-phase injection characterizations are summarized in Supplemental Table 4.

For the 4 improve-phase centers, the aggregated adjusted measure-phase infiltration rate was 8.9% (SE, 3.4%; 95% confidence interval, 4.2%–18.2%). The aggregated adjusted improve-phase rate was 4.6% (SE, 1.9%; 95% confidence interval, 2.1%–10.0%) (Table 2). The difference in rates between the improve phase and the measure phase was 4.3 percentage points, a 48% reduction. The test of H_0_ yielded a *P* value of less than 0.0001, indicating that the overall improve-phase infiltration rate was significantly lower than the overall measure-phase rate.

**TABLE 2:** Unadjusted Measure- and Improve-Phase Infiltration Rates

Center	Measure-phase rate	SE	Improve-phase rate	SE	Change
A	13.3%	2.1%	2.9%	1.0%	−78%
B	15.7%	4.0%	6.0%	2.6%	−62%
C	12.8%	1.5%	8.7%	1.3%	−32%
D	2.1%	0.6%	1.9%	0.6%	−10%

Centers’ volumes are not included to help ensure individual center anonymity.

### Secondary Endpoints

The all-phase factors most likely to be associated with infiltration were nonantecubital fossa injection locations, radiotracer dose, flush volume, and patient weight (Table 3). The rate of reduction at improve-phase centers ranged between 10.0% and 78.4% (median, 46.6%) (Table 2). Improvement plan adherence was high at center A, moderate/low at center B, moderate at center C, and low at center D. A detailed adherence review is found in Supplemental Table 1. Using data from all phases, the variation in infiltration rate at the center or technologist level was significant (*P* < 0.0001 and *P* = 0.0020, respectively).

**TABLE 3:** Associative Factor Analysis for Binary Infiltration Outcome: Significant Associations

Effect (all data, all phases)	*P*
Hand/wrist/forearm injections are associated with higher predicted probability of infiltration than is antecubital fossa injection	<0.0001
Radiotracer dose is positively associated with infiltration	<0.0001
Weight is negatively associated with infiltration	<0.0001
Flush volume is negatively associated with infiltration	<0.0001

### Exploratory Result

Three centers completed a control phase for an average of 22 wk (range, 15.4–25.8 wk) to assess the sustainability of results. This phase was nearly twice the duration of their measure and improve phases and monitored approximately twice as many injections. All centers improved their unadjusted infiltration rates during the control phase, as compared with the measure and improve phases. The aggregated control-phase adjusted infiltration rate was 5.2% (Table 4). The test of H_0_ yielded a Tukey-adjusted *P* value of less than 0.0001, indicating that the control-phase infiltration rate was significantly lower than the measure-phase rate. The test of H_0_ yielded a Tukey-adjusted *P* value of 0.55, indicating that the control phase was not significantly different from the improve phase.

**TABLE 4:** Sustainability at 3 Centers (Control Phase) Using Aggregated Rates

Phase	Adjusted 3-center aggregated infiltration rate	Number of injections	SE	95% CI
Measure	12.1%	815	2.4%	8.2, 17.5
Improve	6.2%	830	1.4%	3.9, 9.5
Control	5.2%	1,763	1.1%	3.5, 7.8

CI = confidence interval.

## DISCUSSION

PET/CT is a sensitive imaging modality with respect to cancer (16,17). Oncologists use PET/CT images to help diagnose and stage disease, choose and plan therapy, and assess tumor response or longitudinally monitor patients (1,18). PET/CT is also used in other clinical applications. Injection infiltration can reduce the sensitivity of PET/CT (19), understate SUVs (4,8,13,15,20,21), and cause other imaging issues. An initial literature review of PET/CT injections for oncology and other clinical applications found that infiltration has negatively affected, or can negatively affect, patient management (Supplemental Table 5) (22–38).

In health-care settings in which infiltration causes acute patient harm, injections are routinely monitored, infiltration is detected and reported, and injection results are assessed by accrediting organizations. In these settings, QI efforts have caused infiltration rates to decline to very low levels, yet clinicians continue to make large-scale efforts to drive rates even lower. Chemotherapy infiltration rates in the 1980s and 1990s ranged from 3% to 6% (5). A recent infiltration benchmarking attempt assessed 739,832 patients and reported a 0.1% chemotherapy infiltration rate (infiltration rates for peripheral intravenous and central venous access devices were estimated at 0.18% and 0.01%, respectively) (6). A 1991–2007 review of studies on infiltration of nonionic iodinated CT contrast media revealed an average rate of 0.45% (39). In 2015, a national data registry and practice quality improvement initiative involving 454,497 CT scans showed that rates had improved to 0.24% (7).

Our literature review found no such large-scale efforts to improve nuclear medicine injections. Our project confirmed that using new technology, centers could routinely monitor injections, establish baseline infiltration rates, and determine center-specific factors (Supplemental Fig. 1) that enable QI processes to reduce PET/CT injection infiltration rates.

The QI project design had its strengths and limitations. The multicenter approach monitored 5,541 injections, nearly double the previously published number of monitored injections. The project demonstrated injection QI across diverse provider types with different practices, different patient volumes, and technologists of varying experience. The project’s prospective nature was also a strength, leading to improved injection processes by using standardized methods to establish infiltration rates, collecting factors associated with injections, and providing individual injection quality control.

The project had limitations. Use of the Lara device added 30 s to the PET/CT procedure for patients and added 90 s/patient to the technologists’ workloads (applying and removing the sensors, providing injection and patient variables). Center representation was a limitation. Five centers supported either academic or NCI-designated comprehensive cancer programs, which comprise 18% of U.S. cancer programs but represented 94.5% of the project’s measure-phase injections (Supplemental Table 6). The other 2 centers supported community providers, and no Veterans Administration centers joined the project. Not collecting information on injection volume, a potential factor associated with infiltration, was also a limitation. Data on the radiotracer injection volume should be captured in future radiotracer QI projects to further examine the association between dose and infiltration rate. The decisions of 3 centers were not contingent on measure-phase results, because these 3 centers did not move beyond the measure phase: one of these centers was replacing PET/CT scanners but remains interested in the analyze and improve phases, the second center transitioned providers, and the third center cited time constraints that prevented moving on. Although the overall rate of injection use was high, lack of 100% use was also a project limitation. Finally, the trial/observer effect was evident throughout the project. Technologists were reminded of the importance of high-quality radiotracer injections; as a result, it is possible that this trial/observer effect contributed to higher-quality injections.

The combination of trial/observer effect, less than 100% use, and overrepresentation of academic centers and cancer programs suggests that the reported measure-phase rates are likely less than the actual incidence of PET/CT injection infiltration in the United States. The lack of 100% use likely biased the tests of improve-phase and measure-phase differences toward null; 100% use would likely have resulted in more pronounced differences.

The project has implications for practice and studies in the field. In the current clinical setting, quality control measures require that an accurate dose be administered to patients (40). On the basis of our findings and the published infiltration rates, it is important to add a quality control measure that ensures the entire dose enters circulation. Not all infiltrations will make a difference to patient care, but some will. Just as patient glucose level, syringe residual, and the time of imaging after injection are monitored and reported today, providing injection-process quality control and including this information in PET/CT reports may prove useful. In addition, since the system can be used for different radiotracer energy levels, a QI methodology could be used to improve some of the 15.5 million annual γ-camera scan injections in the United States (1). Many characteristics associated with PET/CT injections (technologists, patients, technique, and lack of feedback) also exist in γ-camera dose injections. Infiltrated γ-camera procedure injections can also negatively affect patients (41).

Preventing infiltrated injections will become even more important as use of nuclear medicine procedures grows (1,2,42). As efforts are implemented to lower radiotracer doses to as low as reasonably achievable, the infiltrate volume will represent a higher proportion of the administered dose. Finally, the growing use of α- and β-emitting therapeutics is notable. Whereas infiltration of diagnostic radiotracers can result in indirect negative effects on patients, infiltration of therapeutic radiopharmaceuticals may cause acute and severe patient harm (43).

Large studies on radiotracer injections, similar in scale to studies on chemotherapy and contrast CT injections, are needed to provide insight into the frequency and consequences of nuclear medicine infiltration. Such studies may identify factors clearly associated with infiltration and lead to guideline standards that improve injection quality. Nuclear medicine technologist schools could adopt these findings to train future technologists. Additionally, studies into the effect that infiltration has on image quantification could provide tools that guide clinicians on whether to reschedule imaging or proceed with imaging infiltrated patients.

## CONCLUSION

To realize the full diagnostic potential of radiotracer imaging, it is important to perform PET/CT and γ-camera scanning in such a way as to obtain images of the highest quality. Minimizing low-quality radiotracer injections could improve the accuracy and reproducibility of nuclear medicine. This project demonstrated that nuclear medicine infiltration rates can be reduced and sustained through QI. Ongoing monitoring of nuclear medicine injection processes will help ensure that they remain well controlled or continue to improve, just as contrast CT and chemotherapy injection process have continued to improve. Certified nuclear medicine technologist training programs and accrediting organizations might consider adopting injection monitoring as part of their effort to improve the quality and repeatability of PET/CT and other nuclear medicine scans.

## DISCLOSURE

This material is based on work supported in whole or part by the North Carolina Biotechnology Center. Lara devices were provided by Lucerno Dynamics free of charge for the duration of the QI project. Steven Perrin, Kelley Ryan, and Ronald Lattanze are employees of Lucerno Dynamics. Thad Benefield is an employee of UNC Chapel Hill, which received statistical support funding. Dustin Osborne received nonfinancial support from Lucerno Dynamics and is engaging in ongoing research discussions and collaborations outside the submitted work. Terence Wong, James Crowley, and Jackson Kiser receive personal fees from Lucerno Dynamics outside the submitted work. David Townsend holds a PET/CT patent with royalties paid. No other potential conflict of interest relevant to this article was reported.
